# Efficacy of a Novel Ablation Index Algorithm for Cavotricuspid Isthmus Ablation Using a Flexible‐Tip Ablation Catheter

**DOI:** 10.1002/joa3.70378

**Published:** 2026-05-27

**Authors:** Koji Sudo, Kenji Kuroki, Tetsuya Asakawa, Kazuya Nakagawa, Jumpei Saito, Chisa Asahina, Takeaki Goto, Maoko Atsumi, Tomoaki Hasegawa, Yasuteru Yamauchi, Kazutaka Aonuma, Akira Sato

**Affiliations:** ^1^ Department of Cardiovascular Medicine University of Yamanashi Chuo Japan; ^2^ Department of Cardiology Yamanashi Kosei Hospital Yamanashi Japan; ^3^ Department of Cardiology Mito Saiseikai General Hospital Mito Japan; ^4^ Department of Cardiology Yokohama City Minato Red Cross Hospital Yokohama Japan

**Keywords:** atrial flutter, automark index, catheter ablation, cavotricuspid isthmus, radiofrequency ablation

## Abstract

**Introduction:**

AutoMark Index (AI) is a novel ablation algorithm integrating contact force (CF), radiofrequency (RF) current, and application duration to provide real‐time lesion formation feedback. Data of AI using a flexible‐tip RF ablation catheter for cavotricuspid isthmus (CTI) ablation remain limited. The objective was to evaluate the efficacy and safety of AI‐guided CTI ablation.

**Methods:**

Consecutive 148 patients undergoing CTI ablation with a CF‐sensing flexible‐tip ablation catheter were included. Outcomes using an AI‐guided protocol (50 W, targeting AI of 50; AI‐50 group, 62 patients) were compared with a conventional protocol (40 W for 20 s; 40‐W group, 86 patients).

**Results:**

The first‐line success rate showed no difference between the two groups (40‐W group: 72/86 [84.7%] vs. AI‐50 group: 51/62 [82.2%], *p* = 0.81). The total RF application showed no difference (40‐W group: 12 [9.5–15] vs. AI‐50 group: 12 [9.7–16], *p* = 0.50); however, significant differences were observed in the total RF duration (40‐W group: 274 [211–372] s vs. AI‐50 group: 180 [138–249] s, *p* < 0.001), the RF duration for initial block (40‐W group: 225 [163–323] s vs. AI‐50 group: 149 [109–221] s, *p* < 0.001), and CTI procedural time (40‐W group: 9.2 [6.0–14.6] min vs. AI‐50 group: 6.3 [4.5–9.7] min, *p* = 0.007). No significant differences were observed in the mean CF, average temperature, and CTI length between the two groups. No major complications were observed in either of the groups.

**Conclusion:**

AI‐guided CTI ablation protocol is efficient in shortening RF duration for acute bidirectional CTI block and CTI procedure time compared to the conventional 40 W ablation protocol.

## Graphical abstract

AutoMark Index (AI) is a novel ablation algorithm that combines contact force, radiofrequency current, and application to provide real‐time lesion formation. AI‐guided CTI ablation protocol is efficient in shortening RF duration for acute bidirectional CTI block and CTI procedure time compared to the conventional 40 W ablation protocol.

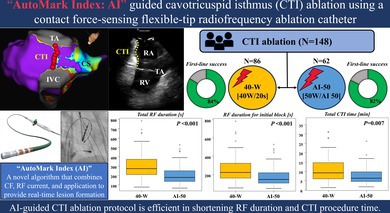

**Abbreviations**
AF: atrial fibrillation
AFL: atrial flutter
AI: autoMark Index
CA: catheter ablation
CTI: cavotricuspid isthmus
ICE: intracardiac echocardiography
LSI: lesion size index
RF: radiofrequency
RFA: radiofrequency ablation

## 1 Introduction

Cavotricuspid isthmus (CTI)‐dependent atrial flutter (AFL) is a common atrial arrhythmia that includes a reentry circuit along the tricuspid annulus (TA) and is an established target for radiofrequency ablation (RFA) and standard treatment [1, 2]. CTI‐dependent AFL frequently coexists with atrial fibrillation (AF), and these arrhythmias are mechanistically linked [3]. Concomitant CTI ablation at the time of pulmonary vein isolation (PVI) reduces early AFL recurrence [4]. The lesion size index (LSI) using a 3.5‐mm open‐irrigated‐tip contact force (CF)‐sensing ablation catheter (TactiCath Sensor Enabled; Abbott, MN, USA) predicts the lesion size for radiofrequency (RF) applications by combining three indices: CF, current, and time. Animal experimental models have shown that LSI can accurately predict lesion size [5]. Recently, a CF‐sensing flexible‐tip irrigated ablation catheter (TactiFlex Sensor Enabled; Abbott) has improved the irrigation efficacy while preserving the lesion size and depth, and is becoming the standard tool for AF ablation. Flexible tip designs have been shown in preclinical studies to enhance tip‐tissue stability. Moreover, the ability to flex and direct irrigation flow to the tip‐tissue interface enhances cooling. It promotes a higher RF power delivery for more effective lesion creation while minimizing the risk of overheating [6]. However, limited data are available that focus on a direct comparison of the performance of different novel irrigation‐tip catheters in human CTI ablation. A CF‐sensing flexible‐tip ablation catheter is now available, and the previously available LSI is no longer displayed. Therefore, we have had to use several protocols, such as low‐power (30 W), standard‐power (40 W), high‐power (50 W) [7], and force‐time‐integral guidance for PVI, owing to the lack of an ablation indicator such as an LSI or ablation index [8]. The AutoMark Index (AI) module is a novel ablation algorithm that combines the CF, RF current, and application duration to provide real‐time lesion formation feedback in a three‐dimensional (3D) electroanatomical mapping system (EnSite X EP System, Abbott). This study aimed to evaluate the efficacy and safety of AI‐guided CTI ablation protocol compared with the conventional 40 W ablation protocol.

## 2 Methods

### 2.1 Study Population

This study was a single‐center retrospective cohort of 148 consecutive patients undergoing CTI ablation between October 2022 and December 2025 at the Yamanashi University Hospital. CTI ablation was performed to prevent AFL in patients undergoing AF ablation or to treat clinical AFL as documented using a 12‐lead electrocardiogram. The protocol selection criteria were based on the conventional CTI protocol of 40 W for 20 s before the availability of the AI algorithm; gradually, the criteria shifted to the AI‐guided CTI protocol of 50 W targeting an AI value of 50, as the AI algorithm became available. The inclusion criteria were patients aged > 18 years who were scheduled for CTI ablation using RFA. Additionally, patients with AF were scheduled for PVI using RFA or pulsed‐field ablation (PFA). Paroxysmal AF was defined as AF lasting for < 7 days. The exclusion criteria were patients who had previously undergone CTI ablation, and those who had severe valvular heart disease, worsening heart failure, or intracardiac thrombus were excluded.

### 2.2 Ablation Procedure

**FIGURE 1:**
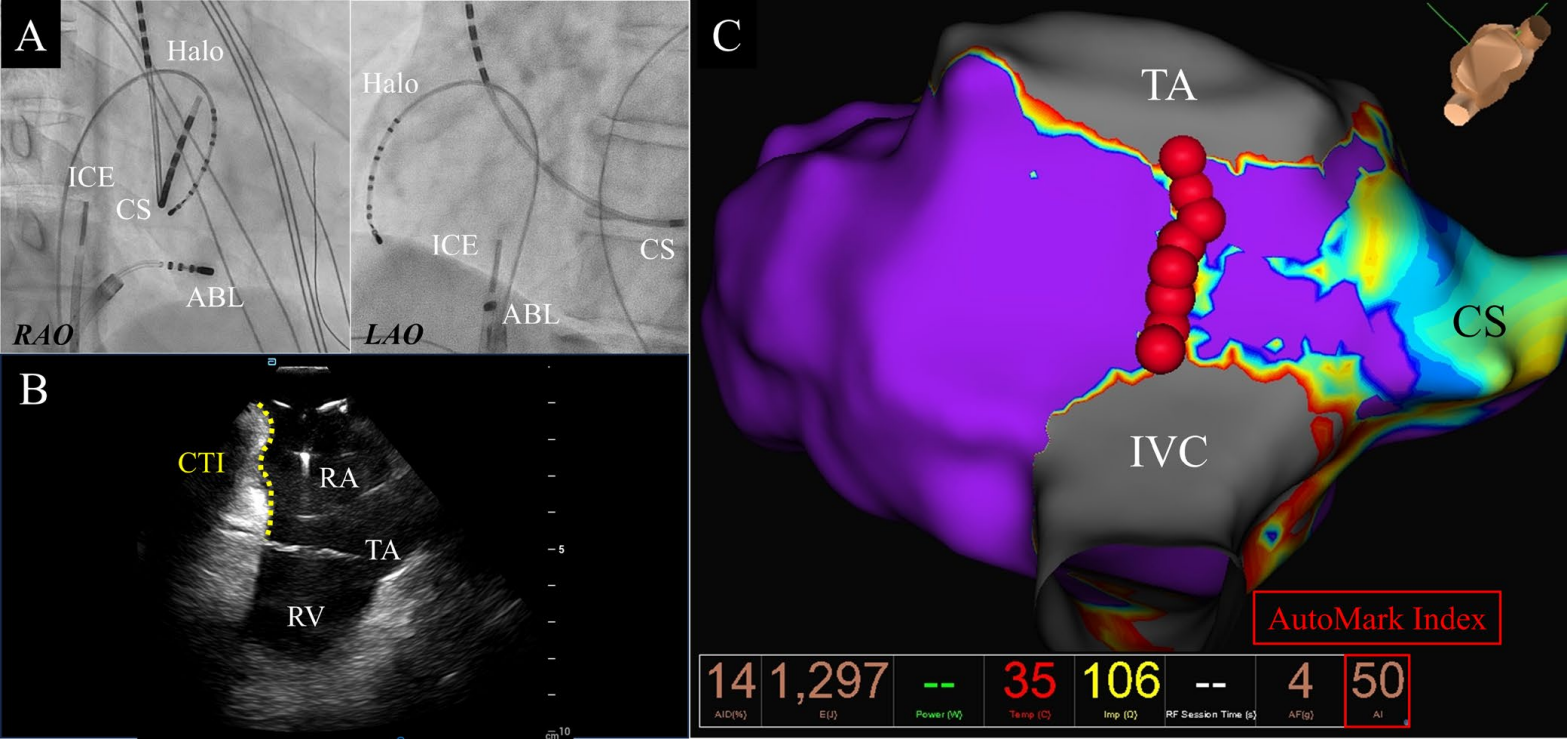
Multimodality for cavotricuspid isthmus ablation. (A) Fluoroscopic images in right and left anterior oblique. (B) Intracardiac echocardiography of cavotricuspid isthmus anatomy. (C) Voltage mapping and geometry from the tricuspid annulus to the inferior vena cava in an inferior view. The target of the AutoMark index was set at 50 (red tags) for the whole segment. AI, Automark index; ABL, ablation catheter; CS, coronary sinus; CTI, cavotricuspid isthmus; TA, tricuspid anulus; ICE, intracardiac echocardiography; IVC, inferior vena cava; RA, right atrium; RV, right ventricular; SVC, superior vena cava.

All procedures were performed using dexmedetomidine for minimal to moderate sedation and fentanyl as an intravenous analgesic. CTI without PVI was performed using pentazocine. Following venous puncture, 5000 units of heparin were administered. Femoral arterial access was performed regularly for continuous arterial pressure monitoring. A 6‐Fr, 20‐pole, 3‐site mapping catheter (BeeAT; Japan Lifeline, Tokyo, Japan) was placed into the coronary sinus (CS) via the right jugular vein for pacing, recording, and internal cardioversion. In patients with AF, PVI was performed using a CF‐sensing flexible‐tip irrigated ablation catheter, which was the same ablation catheter for CTI or PFA. A 3D mapping system with the EnSite X EP system was used for all patients. After transseptal puncture guided by fluoroscopic imaging and intracardiac echocardiography (ICE) (ViewFlex ICE, Abbott), 3000 units of heparin were added, followed by continuous heparin infusion to maintain an activated clotting time of 350–400 s during the procedure. After PVI, a 10‐pole mapping catheter (EP Star, Japan Lifeline) was placed in the lateral right atrium (RA). Subsequently, CTI ablation was performed under electrophysiological, 3D mapping, and fluoroscopic guidance during the CS proximal pacing. The ICE catheter was positioned at the RA to evaluate CTI length between tricuspid annulus (TA) and inferior vena cava (IVC) and anatomical variations such as CTI pouches [9], which sometimes make stable catheter‐tissue contact difficult. ICE images help to reach these pouches or the substrate adjacent to the eustachian ridge (Figure 1). In all groups, ablation was performed from the TA to the IVC by point‐by‐point RF applications. An 8.5‐Fr steerable sheath (Agilis, Abbott) or a 13‐Fr steerable sheath (FaraDrive, Boston Scientific, Marlborough, MA, USA) or a 12‐Fr steerable sheath (FlexCath Contour, Medtronic, Minneapolis, MN, USA) was used to stabilize the ablation catheter. The RF energy setting was 40 W for 20 s in the conventional RF protocol (40‐W group), and the RF setting was 50 W targeting AI of 50 in the novel RF protocol (AI‐50 group). In both groups, the CF was maintained at 5**–**15 g, the temperature limit was < 40°C, and the irrigation rate was 13 mL/min in all ablation points. The inter‐lesion distance was 5 mm in a 3D mapping system. The endpoint of the ablation procedure was the achievement of a bidirectional CTI conduction block. The CTI block was confirmed by differential pacing from the proximal CS and the inferior lateral wall of the RA.

### 2.3 Conduction Gap After CTI Ablation and Additional RF Application

First‐line success was defined that CTI block line was created first pass without gap mapping. In cases in which the first‐line bidirectional CTI block was unsuccessful, the conduction gap was identified through local potentials obtained with the ablation catheter or a high‐density grid mapping catheter (Advisor high‐density grid catheter, Abbott) while maintaining constant pacing from the proximal CS. If a bidirectional CTI block was not achieved, even with a gap site, a new lateral CTI block line was created. Acute success was defined as the persistence of a bidirectional block along the CTI following a 15 min observation period. Total procedure time was defined as the time from groin puncture to sheath removal (skin‐to‐skin time), and CTI procedural time was measured from first RF ablation to block measurements.

### 2.4 Post Catheter Ablation Management

Transthoracic echocardiography was performed on the day after the ablation procedure to exclude pericardial effusion. After the procedure, both anticoagulation and antiarrhythmic drugs were maintained. At the 3 month follow‐up, the decision to continue anticoagulation was based on the patient's stroke risk, while the continuation of antiarrhythmic drugs was left to the treating physician's discretion. Data on periprocedural complications, including vascular complications, cardiac tamponade, stroke, transient ischemic attack, atrio‐esophageal fistulae, phrenic nerve palsy, kidney injury, and mortality, were collected.

All patients were evaluated at the outpatient clinic 1 month after the procedure and subsequently at intervals of 3–6 months. Any atrial tachyarrhythmia defined as episodes > 30 s was considered a recurrence. Assessments with 24 h Holter electrocardiography were performed to detect any arrhythmia recurrences 12 month after the procedure and emergent examination was carried out when patients experienced any symptoms that suggested arrhythmia recurrence.

### 2.5 Statistical Analysis

Categorical variables are presented as numbers and percentages, and continuous variables are presented as the mean ± standard deviation or median with interquartile range (IQR: 25%, 75% percentiles) as appropriate for their distribution. The Student's *t*‐test or the Mann–Whitney *U* test was used to compare the data between the two groups in accordance with the data distribution pattern. Fisher's exact test with 2 × 2 tables was used to compare categorical variables between the two groups. Statistical significance was set at a *p* < 0.05. All analyses were performed using EZR software (Saitama Medical Center, Jichi Medical University, Saitama, Japan), which is a graphical user interface for R (R Foundation for Statistical Computing, Vienna, Austria).

## 3 Results

### 3.1 Patient Population

The study population included 148 consecutive patients (70.1 ± 9.6 years, males 107 [72.2%] patients). Patient characteristics are presented in (Table 1). The mean CHADS_2_ score was 1.6 ± 1.0, and the CHA_2_DS_2_‐VASc score was 2.8 ± 1.3. On preoperative transthoracic echocardiography, the mean left atrial diameter, left atrial volume index, and left ventricular ejection fraction were 41.1 ± 6.8 mm, 42.0 ± 14.3 mL/m^2^, and 61.6% ± 14.8%, respectively. The median brain‐natriuretic peptide value was 95.5 (interquartile range 45.3–169.3) pg/mL. Typical AFL was clinically documented in 18 (12.1%) patients (AFL only: four patients; both AF and AFL: 14 patients) using a 12‐lead electrocardiogram before the procedure. The remaining 130 patients with AF underwent combined AF and CTI ablation without AFL documentation before the procedure. PVI was performed using RFA in 80 patients and PFA in 65 patients (Pentaspline catheter system, 37 patients; loop catheter system, 28 patients). Two ablation protocols were available for CTI ablation: 40‐W group (86 patients) and AI‐50 group (62 patients). No statistically significant differences were observed between the two groups, except for left ventricular ejection fraction, which was higher in the 40‐W group (*p* = 0.002).

**TABLE 1:** Clinical characteristics of the study population.

	Overall (*N* = 148)	40‐W group (*n* = 86)	AI‐50 group (*n* = 62)	*p*
Age, years	70.1 ± 9.6	70 ± 9.9	70.3 ± 9.3	0.84
Sex, male, *n* (%)	107 (72.2)	61 (70.9)	46 (74.1)	0.66
BMI, kg/m^2^	23.9 ± 3.4	24.0 ± 3.5	23.9 ± 3.3	0.80
Atrial flutter, *n* (%)	18 (12.1)	11 (12.7)	7 (11.2)	0.783
AF, *n* (%)	142 (95.9)	81 (94.1)	61 (98.3)	0.488
Paroxysmal AF, *n* (%)	62 (41.8)	36 (41.8)	26 (41.9)	0.99
Persistent AF, *n* (%)	80 (54.0)	45 (52.3)	35 (56.4)	0.61
CHF, *n* (%)	37 (25.0)	17 (19.7)	20 (32.2)	0.083
Hypertension, *n* (%)	93 (62.8)	56 (65.1)	37 (59.6)	0.49
Diabetes mellitus, *n* (%)	34 (22.9)	21 (24.4)	13 (20.9)	0.62
Stroke/TIA, *n* (%)	10 (6.7)	9 (10.4)	1 (1.6)	0.063
CHADS2 score	1.6 ± 1.0	1.6 ± 1.0	1.5 ± 1.0	0.285
CHADS2 VASc score	2.8 ± 1.3	2.9 ± 1.9	2.6 ± 1.3	0.301
Cr, mg/dL	0.92 [0.76–1.13]	0.90 [0.74–1.13]	0.93 [0.81–1.11]	0.91
eGFR, mL/min/1.73m^2^	60 [48–71]	59.5 [48–72.5]	60 [47.5–70]	0.73
BNP, pg/mL	95.5 [45.3–169.3]	89 [35.4–144.8]	102.2 [52.7–206]	0.26
LAD, mm	41.1 ± 6.8	40.8 ± 6.9	41.6 ± 6.5	0.50
LAVI, mL/m^2^	42.0 ± 14.3	41.8 ± 14.5	42.3 ± 14.2	0.83
LVEF, %	61.6 ± 14.8	64.7 ± 12.6	57.3 ± 16.5	0.002

*Note:* Values are presented as mean and standard deviation, median [interquartile range], or number (percentage), as appropriate.

Abbreviations: AF, atrial fibrillation; AI, AutoMark Index; BMI, body mass index; BNP, B‐type natriuretic peptide; CHF, congestive heart failure; Cr, serum creatinine; LAD, left atrial dimensions; LAVI, left atrial volume index; LVEF, left ventricular ejection fraction; TIA, transient ischemic attack.

### 3.2 Procedural Characteristics

**FIGURE 2:**
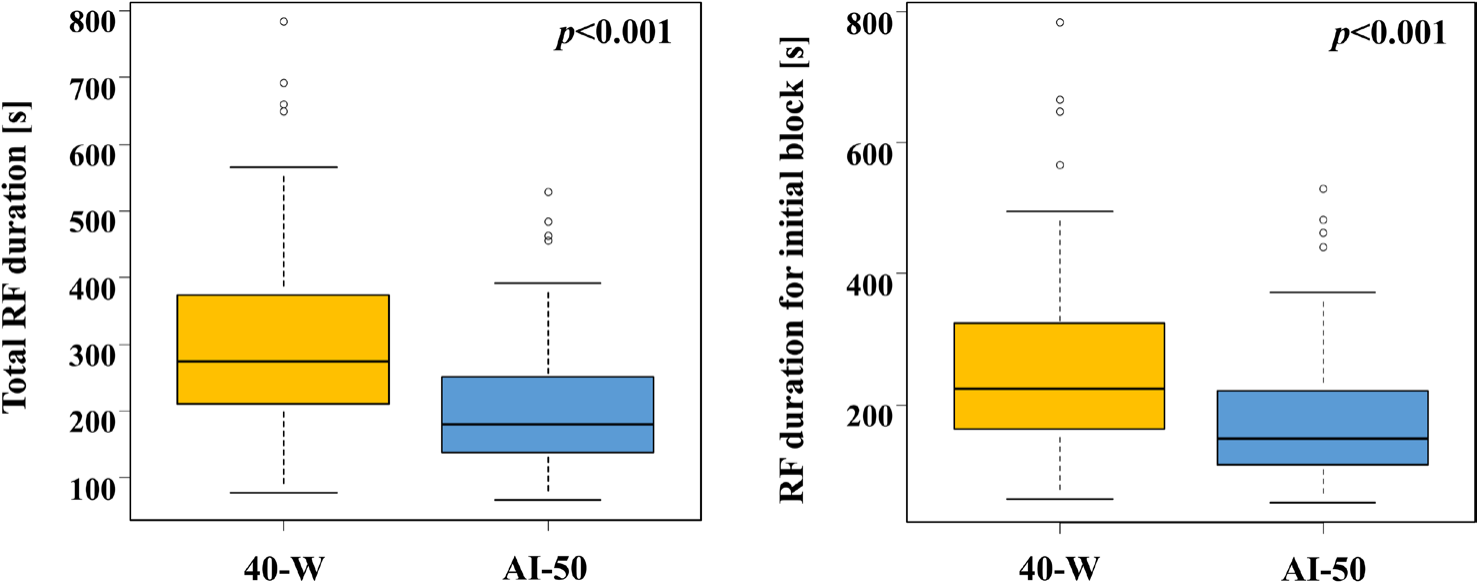
Comparison of radiofrequency duration for cavotricuspid isthmus ablation. Comparison of total RF duration (left panel), RF duration for initial block (right panel) in both the 40‐W group and AI‐50 groups. AI, AutoMark Index; RF, radiofrequency.

**FIGURE 3:**
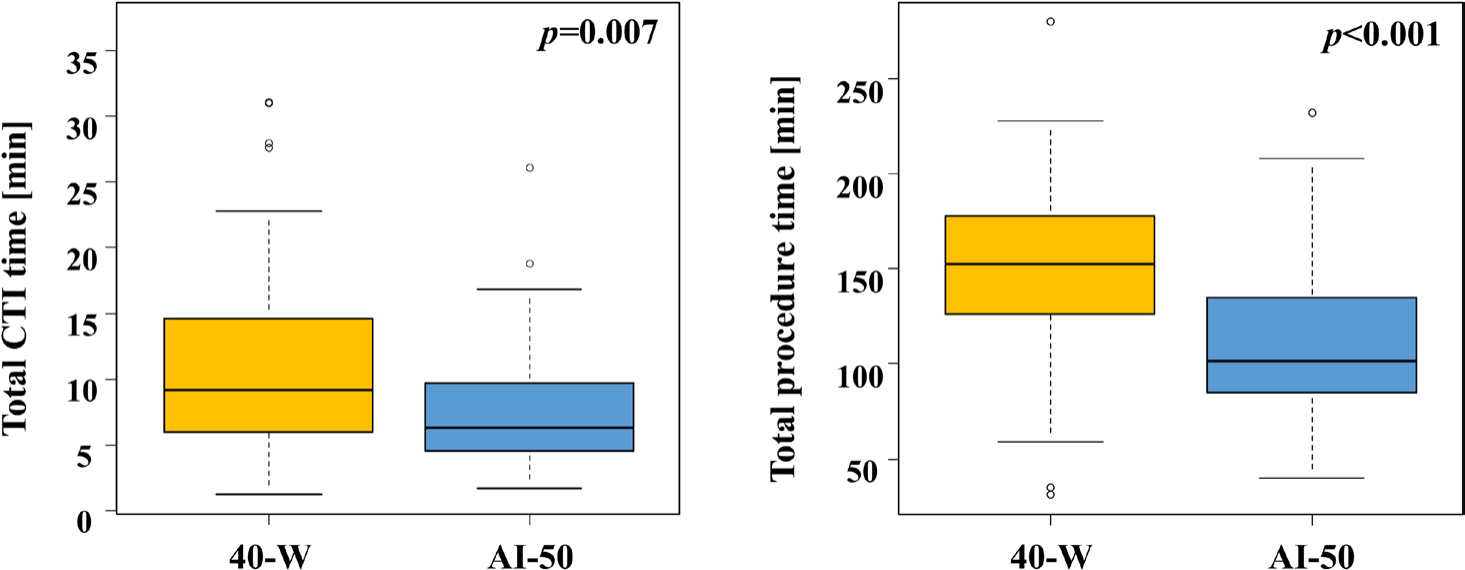
Comparion of procedure time Comparison of CTI procedure time (left panel), and total procedure time (right panel) in both 40‐W group and AI‐50 groups. AI, AutoMark Index; CTI, cavotricuspid isthmus.

Procedural data are summarized in (Table 2). An acute success of CTI bidirectional block line was successfully created in all patients in both groups. The first‐line success rate showed no difference between the two groups (40‐Wgroup: 72/86 [84.7%] vs. AI‐50 group: 51/62 [82.2%]; *p* = 0.81). The catheter inversion technique was same requirement between two groups (40‐Wgroup: 41/86 [47.6%] vs. AI‐50 group: 26/62 [41.9%]; *p* = 0.48). The mean RF duration per application was 22 (20–25) s in the 40‐W group and 14 (13–16) s in the AI‐50 group (*p* < 0.001). Mean AI value was 51.5 ± 2.8 in the AI‐50 group. No differences were observed in the total RF application (40‐W group: 12 [9.5–15] vs. AI‐50 group: 12 [9.7–16]; *p* = 0.50) or in the RF application for the initial block (40‐W group: 9 [7−13] vs. AI‐50 group: 10 [7–13.2]; *p* = 0.37). In contrast, significant differences were observed in the total RF time (40‐W group: 274 [211–372] s vs. AI‐50 group: 180 [138–249] s, *p* < 0.001) and in the RF time for the initial block (40‐W group: 225 [163–323] s vs. AI‐50 group: 149 [109–221] s, *p* < 0.001) (Figure 2). Whereas, 14/86 (16.7%) patients in the 40‐W group and 11/62 (17.7%) patients in the AI‐50 group could not be created CTI block line by first‐line. Among the cases in which first‐pass success was not achieved, there was no significant difference between the two groups in cases in which a gap remained in the IVC (40‐W group: 8/14 (57.2%) vs. AI‐50 group: 7/11 (63.6%), *p* = 0.74) or in cases in which a gap remained in the TA (40‐W group: 2/14 (14.2%) vs. AI‐50 group: 1/11 (9.1%), *p* = 0.69). There was also no significant difference between the two groups in cases in which a new additional line was created (40‐W group: 4/14 (28.6%) vs. AI‐50 group: 3/11 (27.3%), *p* = 0.94). Significant difference was observed in the CTI procedure time (40‐W group: 9.2 [6.0–14.6] min vs. AI‐50 group: 6.3 [4.5–9.7] min, *p* = 0.007), and total procedure time (40‐W group: 152 [127–177] min vs. AI‐50 group: 101 [85–133] min, *p* < 0.001) (Figure 3). No significant differences were observed in the mean CF, average temperature of the ablation catheter, or CTI length between the two groups. Furthermore, no significant difference was observed between the two groups in terms of gap sites. In the 40‐W group, one patient had minor bleeding at the puncture site, but blood transfusion was not required. No audible steam pop was observed during the procedure. No major complications, such as pericardial effusion or cardiac tamponade, were observed.

**TABLE 2:** Procedural characteristics.

	40‐W group (*n* = 86)	AI‐50 group (*n* = 62)	*p*
First‐line success, *n*	72 (84.7)	51 (82.2)	0.81
Acute success, *n*	86 (100)	62 (100)	1
Catheter inversion technique, *n*	41 (47.6)	26 (41.9)	0.48
Total RF application, *n*	12 [9.2–15]	12 [9.7–16]	0.5
Total RF duration, s	274 [211–372]	180 [138–249]	< 0.001
RF duration per application, s	22 [20–25]	14 [13–16]	< 0.001
RF application for initial block, *n*	9 [7.0–13]	10 [7.0–13.2]	0.37
RF duration for initial block, s	225 [163–323]	149 [109–221]	< 0.001
Average CF, g	9.9 ± 2.9	9.3 ± 2.5	0.18
Total RF energy delivery, J	10 359 [8329–14 474]	8276 [6378–11 397]	0.001
Average temperature, °C	33.6 ± 2.0	33.0 ± 1.5	0.055
CTI length, mm	31.0 [27.0–38.2]	33.0 [27.2–37.0]	0.95
Mean AI	—	51.5 ± 2.8	—
Gap site and additional RF site, *n*	14 (15.3)	11 (17.8)	0.81
IVC side, *n*	8 (57.2)	7 (63.6)	0.74
TA side, *n*	2 (14.2)	1 (9.1)	0.69
Additional line creation, *n*	4 (28.6)	3 (27.3)	0.94
CTI procedural time, min	9.2 [6.0–14.6]	6.3 [4.5–9.7]	0.007
PVI system			
RFA, *n*	75 (87.2)	5 (8.0)	< 0.001
PFA, *n*	9 (10.5)	55 (88.7)	< 0.001
Total procedural time, min	152.5 [127–177.5]	101.5 [85–133.2]	< 0.001
Steam pop, *n*	0	0	1
Major complication, *n*	0	0	1

*Note:* Values are presented as mean and standard deviation, median [interquartile range], or number (percentage), as appropriate.

Abbreviations: AI, AutoMark Index; CF, contact force; CTI, cavotricuspid isthmus; IVC, inferior vena cava; PFA, pulsed‐field ablation; PVI, pulmonary vein isolation; RF, radiofrequency; TA, tricuspid annulus.

After a mean follow‐up period of 495 (IQR, 355**–**850) days, 13 of the 148 (8.8%) patients developed atrial arrhythmias (atrial tachycardia; three patients, AF; 10 patients); seven of the 13 (53.8%) patients underwent redo procedures, and bidirectional CTI block line was already preserved and simply confirmed during the redo procedures in all seven patients (40‐W group: four patients, AI‐50 group: three patients).

## 4 Discussion

### 4.1 Major Findings

This study investigated the efficiency and safety of AI, a novel RF algorithm, using a CF‐sensing flexible‐tip irrigated ablation catheter for CTI ablation. We observed the following: (1) Bidirectional conduction block was achieved in all patients, and the same success rate of first‐line success for CTI ablation was observed between the conventional CTI protocol (40 W for 20 s) and the AI‐guided CTI protocol (50 W, targeting AI of 50). (2) The AI‐guided CTI ablation protocol was efficient for shorter RF duration and CTI procedure time than the conventional CTI protocol. (3) The AI algorithm, which targets AI of 50 with 50 W, could be a novel RF protocol for CTI ablation. However, determining the optimal AI setting is still an issue that needs further consideration.

### 4.2 CTI Ablation Setting Using RF

Many clinical reports are available on the use of RF settings for CTI ablation. Previous studies evaluated the efficacy of several types of ablation catheters. They showed that the irrigation‐tip catheters and conventional 8‐mm tip catheters were more effective than non‐irrigated 4‐mm tip catheters for CTI ablation [10, 11]. The 4‐mm flexible‐tip irrigation catheter performed significantly better for CTI ablation than the 3.5‐mm rigid irrigation‐tip catheter [12]. LSI‐guided PVI is associated with improved acute and long‐term success rates compared to CF‐guided catheter ablation [13, 14]. Moreover, Maruyama et al. reported in 2023 that LSI‐guided CTI ablation will be an effective and safe method [15]. Recently, alternative ablation catheters such as a tissue temperature‐controlled catheter have shown efficacy for CTI ablation compared with non‐irrigation and irrigation catheters [16]. Sasaki et al. reported that local impedance measurements could predict effective CTI ablation using a CF‐sensing ablation catheter [17].

### 4.3 Anatomical Characteristics of CTI Using Multimodality Imaging

There have been many reports of various modalities for confirming the CTI anatomy. To ensure sufficient and effective delivery of RF energy for difficult anatomies, such as trabecular bridges and sub‐Eustachian recesses [18], biplane RA angiography has been reported as a useful method for CTI ablation [19]. The ablation procedure is occasionally difficult to perform because of variations in the anatomical characteristics of the CTI. Multidetector row computed tomography has become a widely used substitute for cardiac angiography studies [20]. Furthermore, variable catheter ablation strategies based on preoperative multidetector row computed tomography findings significantly improved the adaptation of the ablation catheter to the anatomical characteristics associated with procedural difficulties in CTI ablation compared to the conventional strategy [21]. When the electrode‐tissue contact was insufficient in the proximal part of the CTI, the catheter inversion technique was useful for maintaining sufficient contact. ICE imaging is useful in CTI ablation for confirming anatomical features [22].

### 4.4 Prospects for CTI Ablation Using PFA

PFA has been introduced as a novel, near nonthermal ablation technology consisting of high‐voltage trains of considerably short pulses, which result in tissue‐specific damage in the heart by cardiomyocyte‐selective electroporation without thermal damage to the surrounding organs [23]. PFA is a safe and effective method for performing PVI in patients with AF [24, 25]. However, PFA for extrapulmonary ablations, such as mitral or CTI ablations, has not yet been well established compared to thermal ablation technology.

### 4.5 Limitations

This study had some limitations. First, this study was a single‐center non‐randomized study with a small sample size, and the RF power setting also differed. The ablation catheter tip size was the same in both groups; therefore, a comparison using the same RF power setting in both groups would have been necessary to more appropriately assess the independent utility of AI‐guided CTI ablation. Therefore, a prospective randomized study is required to demonstrate the efficacy of AI for CTI ablation compared with that of a conventional RF setting of 40 W and the same RF power setting of 50 W. Furthermore, there is still not much data about AI on clinical study, and various AI cutoff values are needed for optimal setting depending on the ablation site. Second, it must be considered that the efficacy and safety of ablation performed clinically depend on factors such as the ablation catheter stability during RF application, the anatomy of the CTI, and the clinical experience of the operator. In this study, only experienced operators engaged in ablation procedures. Third, CTI ablation was mainly performed in patients with AF to prevent common AFL, and we did not evaluate long‐term recurrence of AFL. Therefore, we aim to follow up on the long‐term recurrence of AF and AFL and examine the durability of CTI ablation between the two groups.

## 5 Conclusions

AI is a novel algorithm to provide real‐time lesion formation. Although the first‐line success rate showed no superiority in AI‐guided CTI ablation protocol (50 W, targeting AI of 50), AI‐guided CTI ablation protocol is efficient in shortening RF duration for acute bidirectional CTI block and CTI procedure time compared to the conventional 40 W ablation protocol.

## Ethics Statement

This study was conducted using an opt‐out method. The requirement for obtaining individual informed consent was waived because only de‐identified data were used. Instead, information regarding the study was disclosed on the website of our institution, where patients had the option to decline participation. This study was approved by the Institutional Review Board of the University of Yamanashi (Approval No. R06801).

## Consent

This study was conducted using an opt‐out method.

## Conflicts of Interest

Kenji Kuroki and Akira Sato received honoraria and research grants from Abbott Japan Inc. The other authors declare no conflicts of interest.

## Data Availability

The data that support the findings of this study are available on request from the corresponding author. The data are not publicly available due to privacy or ethical restrictions.
